# Detection of Antibodies against Hepatitis A Virus (HAV) by a Surface Plasmon Resonance (SPR) Biosensor: A New Diagnosis Tool Based on the Major HAV Capsid Protein VP1 (SPR-HAVP1)

**DOI:** 10.3390/s21093167

**Published:** 2021-05-03

**Authors:** Gabriel Menezes Costa dos Santos, Carlos Roberto Alves, Marcelo Alves Pinto, Luciane Almeida Amado Leon, Franklin Souza-Silva

**Affiliations:** 1 Laboratório de Desenvolvimento Tecnológico em Virologia, Instituto Oswaldo Cruz, Fundação Oswaldo Cruz, Avenida Brasil, 4365, Manguinhos, Rio de Janeiro CEP 21040-900, Brazil; gabriel.santos@ioc.fiocruz.br (G.M.C.d.S.); marcelop@ioc.fiocruz.br (M.A.P.); 2 Laboratório de Biologia Molecular e Doenças Endêmicas, Instituto Oswaldo Cruz, Fundação Oswaldo Cruz, Avenida Brasil, 4365, Manguinhos, Rio de Janeiro CEP 21040-900, Brazil; calves@ioc.fiocruz.br; 3 Centro de Desenvolvimento Tecnológico em Saúde (CDTS), Instituto Nacional de Ciência e Tecnologia de Inovação em Doenças de Populações Negligenciadas (INCT/IDPN), Fundação Oswaldo Cruz, Avenida Brasil, 4365, Manguinhos, Rio de Janeiro CEP 21040-900, Brazil; Franklin.souza@cdts.fiocruz.br; 4 Faculdade de Ciências Biológicas e da Saúde, Universidade Iguaçu, Avenida Abílio Augusto Távora, 2134, Dom Rodrigo, Nova Iguaçu CEP 26260-045, Brazil

**Keywords:** hepatitis A virus, major capsid protein VP1, IgM, surface plasmon resonance

## Abstract

Hepatitis A (HA) is an acute human infectious disease caused by a positive single-stranded RNA virus (HAV). It is mainly acquired through the fecal–oral route and is primarily spread by contact between people and exposure to contaminated water and food. Recently, large outbreaks of HA have been reported by low and moderate endemicity countries, emphasizing its importance in public health and the need for rapid and large-scale diagnostic tests to support public health decisions on HA. This work proposes a new tool for HAV diagnosis based on the association of surface plasmonic resonance with major capsid protein VP1 (SPR-HAVP1 assay), detecting IgM antibodies for HAV in human serum samples. Structural analyses of VP1 B-lymphocyte epitopes showed continuous and discontinuous epitopes. The discontinuous epitopes were identified in the N-terminal region of the VP1 protein. Both epitope types in the VP1 protein were shown by the reactivity of VP1 in native and denaturing conditions to IgM anti-HAV, which was favorable to tests of VP1 in the SPR assays. SPR-HAVP1 assays showed good performance in the detection of IgM polyclonal antibody anti-HAV. These assays were performed using a COOH5 sensor chip functionalized with VP1 protein. The sensorgram record showed a significant difference between positive and negative serum samples, which was confirmed by analysis of variation of initial and final dissociation values through time (ΔRUd/t). The data gathered here are unequivocal evidence that the SPR-HAVP1 strategy can be applied to detect IgM antibodies in human serum positive to the HAV. This is a new tool to be explored to diagnose human HAV infections.

## 1. Introduction

Hepatitis A is an acute liver disease caused by the hepatitis A virus (HAV). HAV is classified within the *Picornaviridae* family, in the genus *Hepatovirus*. Its genome is a single-stranded, positive-sense RNA, approximately 7.5 kb in length. A single open reading frame (ORF) encodes a polyprotein, which is processed co-translationally into four structural proteins, VP4, VP2, VP3, and VP1, which form the capsid, and six nonstructural proteins, 2A, 2B, 2C, 3A, 3B, and 3C, which are essential for virus replication. The HAV VP1 protein is the most abundant of the viral capsid and is responsible for the formation of the conformational epitopes of neutralization of the virion together with the VP3 protein [1,2,3].

The virus is mainly acquired through the fecal–oral route and is primarily spread by contact between people and exposure to contaminated water and food. Transmission can also happen through sexual activity, especially in men who have sex with men [4]. Hence, the infection is associated with poor sanitary and hygiene conditions, as well as oral–anal sex.

HA is an acute disease, and fatal outcomes are rare; however, it can cause mild to severe symptoms and fulminant hepatitis (acute liver failure), which is often fatal. The World Health Organization (WHO) estimates that 1.5 million people are infected yearly with HAV [5] and that in 2016, 7134 persons died from hepatitis A around the world (representing 0.5% of the mortality due to viral hepatitis) [6]. Hepatitis A has been spread through numerous outbreaks, particularly associated with contaminated fruits and vegetables [6]. Since 2017, low endemicity countries of Europe and the Americas (Chile and the United States of America) have reported an unordinary increase in cases of hepatitis A, predominantly among men who have sex with men (MSM) [6]. Following these large outbreaks, the importance of laboratory diagnosis to support public health approaches for hepatitis A has been emphasized, expecting that future outbreaks could be better assessed [6].

The current epidemiological scenario of hepatitis A in many countries calls for introduction of new rapid and large-scale methodologies aiming to improve vaccination coverage, specifically within this risk group [7]. The standard diagnosis of acute hepatitis A is based on serum anti-HAV IgM detection [8], which is typically detectable at the onset of symptoms and can persist for up to 2–9 months post-infection. IgG antibody follows IgM response after 1 week and provides life-long protection against [9]. Therefore, a diagnostic assay with high sensitivity for the detection of IgM is a valuable tool for the timely care of patients and for the control of infection during HAV outbreaks.

Currently, the most used technique for serological diagnosis of hepatitis A is ELISA. However, although it plays important roles in HA diagnosis, it has disadvantages related to the long run time, indirect format, laborious procedures, cost, and the requirement for multiple washing steps [10,11]

From this perspective, plasmonic-based biosensors can be a promising alternative to the immunoenzymatic tests currently available by providing cost-effective diagnostic methods and real-time monitoring [12]. Among the biosensor technologies that have emerged in the last decades for virus research, plasmonic resonance applications have triggered significant interest, in view of their versatility, label-free monitoring, and low response time [13,14,15]. Thus, surface plasmon resonance (SPR) biosensors represent a promising approach to reach ultra-low detection limits of antibodies from clinical specimens [16].

This work proposes a new tool for HAV diagnosis based on the association of SPR with major capsid protein VP1 assay (SPR-HAVP1), detecting antibodies for HAV in human serum samples.

## 2. Materials and Methods

### 2.1. Chemical and Reagents

Detergents (Tween 20 and sodium dodecyl sulfate (SDS)), bovine serum albumin (BSA), ProteoSilver™ Kit, diaminobenzidine, β-mercaptoethanol, and horseradish peroxidase-conjugated goat anti-human IgM antibody (IgM HP) were acquired from Sigma-Aldrich Chemical Co. (St. Louis, MO, USA). The HAV viral protein 1 (HAV VP1, recombinant) was acquired from Meridian Life Science (Memphis, Tennessee, USA). Nitrocellulose (0.2 µm), Precision Plus Protein™ Kaleidoscope™ Prestained Protein Standards and electrophoresis reagents were acquired from Bio-Rad Laboratories Inc. (US). Hydrogen peroxide (H_2_O_2_) and glycerol were purchased from Merck Millipore Corporation (Darmstadt, Germany). Amine coupling agents (1-ethyl-3-(3-dimethylaminopropyl) carbodiimide (EDC) and N-hydroxysuccinimide (NHS)) and ethanolamide were purchased from Cytiva (Piscataway, NJ, USA). A carboxylated gold sensor chip (COOH5) was acquired from FortéBio-Sartorius BioAnalytical Instruments (Fremont, CA, USA). All other reagents were of analytical grade or better.

### 2.2. Serum Samples from Patients

Validation of the anti-HAV chip was performed using human sera. Serum samples were collected from five healthy blood donors and from two subjects with acute hepatitis A (CAAE: 48376015.2.0000.5248; Opinion Number: 1610747). Serum samples were tested in triplicate for the presence of anti-HAV IgM antibodies using enzyme-linked immunoassays (ELISA) (Bioelisa HAV IgM, Biokit, Barcelona, Spain) according to the manufacturer’s instructions.

### 2.3. IgM Purification

IgM polyclonal antibody against hepatitis A virus (IgM anti-HAV) was obtained by affinity chromatography using HiTrap^®^ IgM Purification HP column with Sepharose^®^ (GE Life Science, Chicago, IL, USA). The concentration of IgM was evaluated using a NanoDrop (Thermo Scientific NanoDrop One).

### 2.4. HAV Samples in Cell Culture

HAV (strain HAF-203) was obtained by cell culture, described by Villar et al. (2004) [17]. Briefly, fetal rhesus kidney–4 (FRhK-4) cells were grown to confluence. After washing the monolayers with phosphate-buffered saline (0.01 M PBS) (pH 7.2), they were inoculated with HAV strain HAF-203 (3.0 mL) (isolated previously) [18]. The virus was adsorbed for 1 h at 37 °C, and cultures were kept at 37 °C for 7 days. The virus was extracted from the cells by freeze-thawing the bottle contents three times. The collected fluid was sonicated followed by a brief low-speed centrifugation and then was aliquoted and stored at −70 °C.

### 2.5. Prediction of VP1 Protein B-Lymphocyte Epitopes

The amino acid sequence of the VP1 protein was accessed at Protein Data Bank server (http://www.pdb.org/-code PDB 4QPG-accessed on 7 April 2020). The VP1 sequences were analyzed for linear (BCPREDS server—http://ailab.ist.psu.edu/bcpred/index.html) and conformational (DiskTope 2.0 server—http://www.cbs.dtu.dk/services/DiscoTope/ and IEDB analyze resource—http://tools.immuneepitope.org/ellipro) epitopes.

### 2.6. SDS Polyacrylamide Gel Electrophoresis (SDS-PAGE)

The VP1 protein was analyzed by SDS-PAGE under reduced and denatured conditions. The VP1 protein samples (10 µg) were mixed with buffer (*v*/*v*; 80 mM Tris-HCl, pH 6.8, 12% glycerol (*v*/*v*), 2% SDS (*w*/*v*), 5% β-mercaptoethanol (*v*/*v*), and 0.05% bromophenol blue (*w*/*v*)), and after boiling for a duration of 3 min, they were loaded into 12% Bis–Tris gel and run at 150 V, 15 mA for 40 min. The gels were stained with Coomassie Blue stain to visualize proteins, and Kaleidoscope^®^ (Bio-Rad) prestained protein standards were used to monitor the electrophoresis of the assayed VP1 protein. 

### 2.7. Immunoenzymatic Assays

#### 2.7.1. Western Blotting 

After electrophoresis in denaturant conditions, the VP1 protein was transferred to a nitrocellulose membrane [19]. The membrane was incubated (25 °C, 2 h) with blocker buffer PBST-milk (0.5% skimmed milk (*w*/*v*) in PBS, 0.5% Tween 20). After that, the membrane was washed (3-fold) with PBS-Tween 20 (0.05%) (PBST) and incubated (25 °C, 1 h) with IgM anti-HAV diluted in PBST (1:3000). After washing (6-fold, 5 min) with PBST, the membranes were incubated (25 °C, 1 h) with anti-IgM HRP (1:3000). After an additional washing step, the immune complex was revealed with diaminobenzidine in citrate/phosphate buffer, pH 5.0, 30% H_2_O_2_.

#### 2.7.2. Dot Blot

The VP1 protein (5 µg) and HAV (6µL) from cell culture supernatant (viral titer = 5.024 log TCID50/mL) samples were loaded directly on a nitrocellulose membrane in a single spot using a vacuum apparatus. After that, the membrane was incubated (65 °C, 2 h) without any buffer. After this step, it was incubated with blocker buffer (37 °C, 16 h), followed by incubation (25 °C, 1 h, swinging) with IgM anti-HAV in PBST-milk (1:50). After washing (6 × 5 min) with PBST-milk, the membranes were incubated (25 °C 1 h,) with an anti-human HRP (1:1000) and washed (3 × 5 min). The immune complex was revealed as described for *W*estern blotting assay.

### 2.8. Establishment of the SPR-HAVP1 Assays

All analyses were performed using an optical biosensor transduction SensíQ^®^ Pioneer FortéBio—Sartorius BioAnalytical Instruments (Fremont, CA, USA). Detection of anti-HAV IgM was performed as a result of interaction between antibody and recombinant VP1 immobilized onto the sensor chip. The SPR response is proportional to the change in mass concentration onto the sensor chip and expressed as a response unit (RU).

#### 2.8.1. Immobilization of VP1

VP1 (10 μg) was immobilized onto sensor chip COOH5 by using the EDC/NHS method [20]. The sensor chip was functionalized with running buffer (HBS-EP buffer: 10 mM HEPES (*w*/*v*), 3 mM EDTA (*w*/*v*), 150 mM NaCl (*w*/*v*), 0.005% Tween 20 (*v*/*v*), pH 7.4), at a continuous flow rate, as follows: (i) injection of 10 μL of 50 mM HCl (*v*/*v*) at 10 μL/min; (ii) injection of 100 μL of 10 mM CH_3_COOH (*w*/*v*) at 50 μL/min; (iii) injection of 50 μL of the mixture (1:1; 0.4 M EDC and 0.1 M NHS) for 2 min at 50 μL/min; (iv) injection of 150 μL of 100 mM ethanolamine at 20 μL/min; (v)100 μL injection of VP1 at 10 μL/min; and (vii) injection of 150 μL of the HBS-EP.

#### 2.8.2. Standardization of Anti-HAV IgM Binding

The detection of anti-HAV IgM antibodies was assessed after the immobilization step of recombinant VP1 onto the gold surface sensor chip. The guarantee of the binding assay reproducibility between tests was achieved by establishing the conditions for regeneration. Therefore, after the antigen/antibody interaction on the sensor chip, the regeneration solution (50 μL of 0.2 M glycine, pH 1.5) was injected at flow rate of 50 μL/min, followed by a new injection cycle with HBS buffer (200 μL, at a flow rate of 10 μL/min). The assays were repeated in triplicate.

Afterward, a calibration curve of concentrations of IgM polyclonal antibody anti-HAV at a concentration range of 3.5 nM to 0.02 nM in serial dilution (factor 2×, 10 μL/min for 10 min) was plotted, interspersed by the regeneration step. The change in response is proportional to the change in mass at the surface and expressed as the unit of response (RU). The association and dissociation sensorgrams for complex formation were obtained after analysis in the SPR Qdat test software (FortéBio, Fremont, CA, USA).

### 2.9. Detection Assay of Anti-HAV Antibody in Serum Samples 

The assays for detection of anti-HAV antibody were performed with different dilutions of human serum samples (1:1000, 1:2000, and 1:5000), positive and negative to anti-HAV. The BSA was assessed at the same concentration range, as a negative control of the binding. All assays were performed in the presence of HBS buffer (100 μL) at a flow rate of 10 μL/min, in triplicate. The linearity range, maximum binding capacity of anti-HAV IgM, sensitivity, and coefficient of variation (CV) for the assay were determined. 

### 2.10. Data Analysis

SPR data were confirmed by linear regression using the VP1 protein concentrations (nM) for defining the coefficient of determination (R^2^). The RU variation and the RU of dissociation (RUd) values were obtained by VP1 detection. The calibration curve was determined by the ratio of the concentration of IgM anti-HAV (abscissa) to RUd (ordinate) resulting from the antigen/antibody interaction. These interactions were accessed as follows:

$$\Delta RUd=\frac{RUid-Rufd}{t}:$$

where RUid is the RU Initial dissociation (RUid), RUfd is the RU final dissociation (RUif), and t is the time in seconds.

The t-Student test was considered for analysis of results, and data were accepted as statistically distinct when *p*-value < 0.05. This analysis was performed using GraphPad Prism version 5.03 (GraphPad Software, San Diego, CA, USA).

## 3. Results

### 3.1. In Silico Analysis of the B-Lymphocyte Epitopes Predictions of VP1 

**Figure 1:**
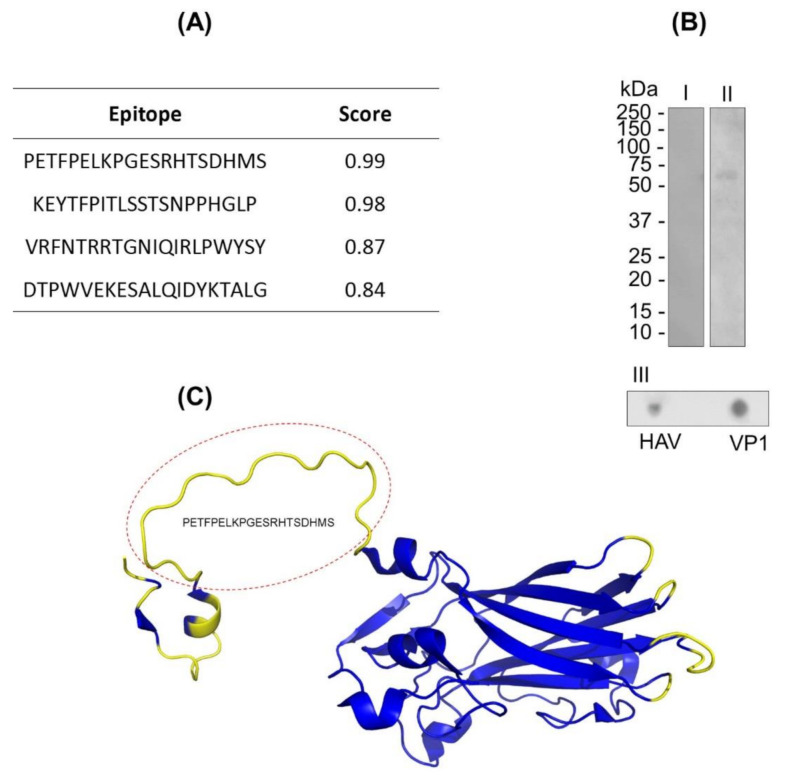
VP1 protein B-lymphocyte epitope characterization. (**A**) VP1 protein epitope prediction using the continuous method by the BCPREDS server. (**B**) Immunoenzymatic assays: Western blot (I and II) and dot blot (III). In this assay, the VP1 protein (10 µg) and hepatitis A virus (HAV; 6 µL) were adsorbed to the nitrocellulose membrane and incubated with anti-HAV IgM-peroxidase. (**C**) VP1 protein epitope prediction by the discontinuous method. The 3D structure of VP1 protein (code: PDB 4QPG) is represented by alpha helix, beta sheet, and coils. The predicted region to discontinuous epitopes is in yellow. The predicted region highlighted in the red circle corresponds to the PETFPELKPGESRHTSDHMS peptide, which is simultaneously predicted as a continuous and discontinuous epitope.

The B-lymphocyte epitopes’ predictions of VP1 protein were assessed by in silico assays using BCPREDS and DiskTope servers. In the characterization of continuous and discontinuous epitopes of the VP1 protein through in silico prediction, four epitopes were identified (Figure 1A). Based on the physicochemical properties of the VP1 sequence, the BCPRED server algorithm was able to predict four peptides (position) with properties to stimulate B lymphocytes in the score range from 0.83 to 0.99 (Figure 1A). The prediction of discontinuous epitope regions was mostly identified in the N-terminal region of the VP1 protein (Figure 1B). We emphasize that both predictions identified the common sequence Nt-PETFPELKPGESRHTSDHMS-Ct regarding the properties of containing continuous and discontinuous epitopes (Figure 1B).

### 3.2. In Vitro Analysis of Continuous and Discontinuous Epitopes in VP1

The continuous and discontinuous epitopes in VP1 protein were also assessed by in vitro assay by using IgM anti-HAV. The reactivity of VP1 in native and denaturing conditions to IgM anti-HAV was proven by dot blot and Western blot assays, respectively, confirming the occurrence of both types of epitopes (Figure 1C). In addition, the specificity of IgM polyclonal recognition against the viral particle was confirmed in the dot blot tests (Figure 1C).

### 3.3. SPR Assays to Detect Anti-VP1 Immunoglobulin

#### 3.3.1. Sensor Chip Stability and Regeneration

**Figure 2:**
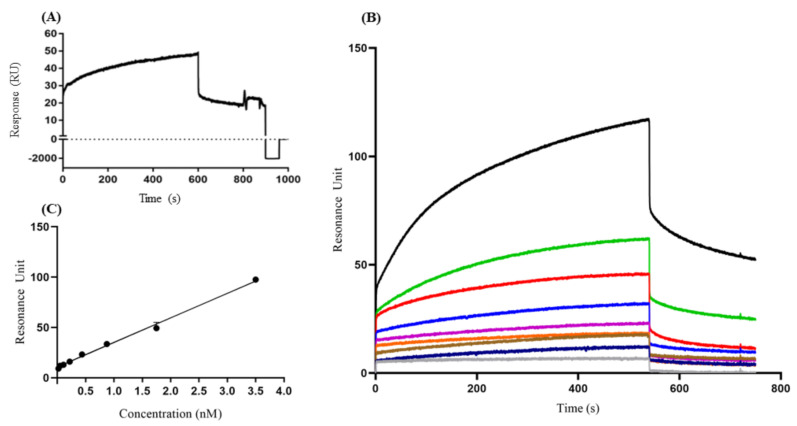
Detection profile of VP1 protein by anti-HAV IgM through the surface plasmonic resonance assays. (**A**) Sensorgram regeneration of IgM binding with VP1 protein immobilized on the chip after 1 min of injection with glycine buffer (**B**) Sensogram of the anti-HAV IgM concentration kinetics (gray HBS-EP, blue 0.02 nM, brown 0.05 nM, orange 0.109 nM, purple 0.218 nM, dark blue 0.437 nM, red 0.875 nM, green 1.75 nM, and black 3.5 nM), (**C**) The data of the dissociation resonance units (RUd) and protein concentration (μg) were analyzed by linear regression. The results are presented as resonance units (RU) response between 1 and 1000 (**A**) and 1 and 800 (**B**) s. These results are representative of four independent assays.

The stability of the VP1 on the sensor chip surface was evaluated by repeated injections of anti-HAV IgM (1.75 nM) and surface regenerations. For this, the binding profile was evaluated in a dose–response curve of anti-HAV IgM with VP1 immobilized in the sensor chip. The sensorgram of regeneration indicated RU response values below IgM anti-HAV/VP1 interaction values at the end of 60 s after the injection with glycine buffer (Figure 2A). From these data, it was possible to state that the VP1 protein remained on the chip, and both continuous and discontinuous B-lymphocyte epitopes remained free for a new interaction (Figure 2B).

#### 3.3.2. Interaction of IgM anti-HAV Antibodies with VP1

The binding property of VP1 to the specific IgM anti-HAV was accessed, demonstrating an increase of RUd signal per second, indicating the concentration-dependent dose of IgM anti-HAV to VP1 (Figure 2B,C). A calibration curve was evaluated plotting anti-HAV IgM at a concentration range of 0.02 nM to 3.5 nM. However, in the dissociation phase, only concentrations of 0.218 nM, 0.437 nM, 0.875 nM, 1.75 nM, and 3.5 nM showed RU values that were concentration-dependent, while concentrations of 0.02 nM, 0.05 nM, and 0.109 nM showed an overlap in the dissociation phase. Therefore, we defined the concentration range with a correlation of coefficient (R^2^) > 0.95 by linear fitting (0.218 nM to 3.5 nM) of anti-HAHV IgM concentration (R^2^ = 0.994). 

The coefficient of variation (CV) of the RU, assessed by evaluation of different concentrations of IgM anti-HAV, varied from 0.4% to 10.8%, illustrating good reproducibility of the assay. Based on these data, it was possible to determine the affinity constant (KD) of idiotype IgM anti-HAV to VP1 protein (KD = 1.82 nM). In addition, because of the possibility of unspecific binding of VP1 to a globular protein unrelated to the immune response, BSA was also used (Supplementary Figure S1). As a background response, these tests indicated lower RUd values (105 RU) than the response value for the formation of the IgM anti-HAV/VP1 complex, indicating the specificity of the tests.

#### 3.3.3. Assessing the anti-HAV Antibodies in Human Serum Samples by SPR-HAVP1 Assay

To address the efficacy of the SPR-HAVP1 approach adopted here, the potential of the VP1 protein immobilized onto the chip sensor to detect antibodies in serum samples from patients in high titers of IgM anti-HAV was evaluated. The tested group contained five negative samples and two highly positive samples. In the ELISA test, specimens with signal to cutoff (S/CO) values of ≥1.0 were considered reactive for IgM anti-HAV, S/CO values of <0.9 were considered non-reactive, and values of 0.9–1.0 were considered indeterminate, according to the manufacturer’s recommendations (Bioelisa HAV IgM, Biokit, Barcelona Spain).

**Figure 3:**
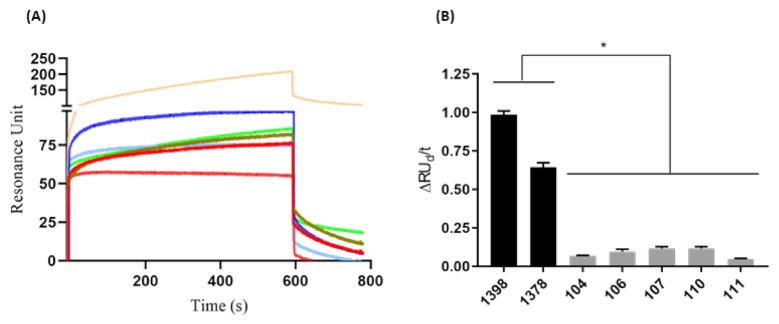
Binding avidity evaluation of anti-HAV for serum samples. (**A**) Human serum (1:1000) positive [1378 (light green) and 1398 (light orange)] and negative [104 (light blue), 106 (red **───**), 107 (dark blue), 110 (green musk), and 111(pink)] from hepatitis A virus. (**B**) The difference between positive (■) and negative (■) serum was analyzed from the variation of the initial and final dissociation values in 177 s (ΔRUd/t). The results are shown as resonance units (RU) and are representative of the average response between 1 and 800 s. These results are representative of three independent assays. * *p* = 0.0006.

The performed tests showed an increase in RU when the positive serum samples for the HAV were directly proportional to the dilution (R^2^= 0.98), showing that a 1:1000 serum dilution gave the best results (Supplementary Figure S2). The SPR assay clearly discriminated between positive and negative samples, since the RU signal in the sensorgram was threefold bigger in positive samples compared to negative samples (Figure 3A). A significant difference (*p* = 0.0006) between positive and negative serum samples was more evident after performing analysis of the variation of initial and final dissociation values in the time of 173 s (ΔRUd/t). 

The initial dissociation phase (RUid = 209.27) and the final dissociation (RUfd = 106.21) of serum 1398 showed higher values than serum 1378 (RUid = 85.66 and RUfd = 19.35). Negative sera had more homogeneous RUid values from 55.14 to 82.25 and RUfd ranging from 1.3 to 13.17, except for serum 111, which presented RUid = 55.16 and did not present positive RUfd. The sensorgram generated from serum samples allowed us to evaluate the avidity of each serum sample based on the RU variation of dissociation divided by time. In this way, it was possible to determine a cutoff value for discrimination between positive (≥0.25) and negative (≤0.15) serum samples (Figure 3B). Additionally, the CV generated by the repeated injections of serum samples (triplicate) onto the chip sensor functionalized with VP1 was found to be from 1.15% to 6.86%, indicating high reproducibility of the assay.

## 4. Discussion

The specific diagnosis of acute hepatitis A depends on the detection of serum IgM antibody to HAV [14]. Currently, this diagnosis is mainly based on ELISA and chemiluminescence immunoassays. Although these assays show good sensitivities and can be automated, they are not high-throughput assays and do not allow large-scale testing [8]. Most of these immunodiagnostic tests for anti-HAV detection rely on the use of inactivated HAV particles as a tool for antibody detection [21]. However, HAV grows slowly and produces low titers in most cell culture systems [22,23], a feature that hampers its mass production for diagnostic tests. Difficulties in producing HAV by cell culture may be circumvented by the use of well-defined antigens. Alternatively, the use of recombinant VP1 proteins may overcome this issue to obtain large amounts of antigen in a faster and cheaper approach, for application in diagnostic tests for HA. Thus, this work explores, for the first time, the association of the recombinant VP1 with SPR technology as a new tool (SPR-HAVP1) for HA diagnosis.

The immunodominant neutralization site of HAV mainly involves residues of VP1 and VP3 and a potentially independent site involving residue 221 of VP1 [24]. Due to the recognized role of VP1 in the humoral immune response during infection, this protein has been the main target of interest for application in the diagnosis of hepatitis A [21,25]. The identification of B cell epitopes in target antigens is one of the crucial steps in immunodiagnostic tests based on epitope [26]. In silico prediction of B-lymphocyte epitopes of the VP1 protein in this study was an important and cost-effective approach, allowing the identification of possible antibody binding sites to this protein. This was one of the advantages of this approach that proved the antigenic potential of VP1 and the functionality of the proposed SPR methodology, adding confidence to the tests with the biosensor in the detection of antibodies directed to the linear and conformational epitopes present in VP1, as shown here by B-lymphocyte epitope prediction, and to both of the performed enzymatic assays, Western blot and dot blot.

In the present work, the SPR-HAVP1 technique was described for the first time for anti-HAV IgM detection. In immunoassays, immobilization of the highest possible amount of antigen is commonly critical to guarantee the greatest antibody binding and to increase the sensitivity and stability of the antigen–antibody complex on the surface of the sensor chip [27]. In this study, using the standard amine coupling procedure, the optimal immobilization of the VP1 was shown to occur. We have evidence that 0.2 M glycine buffer at pH 1.5, followed by a new cycle with HBS buffer was able to remove the bound anti-HAV while maintaining surface activity. Therefore, it was used as a regeneration buffer since it provided consistency in the chip sensor surface activity after several cycles of regeneration.

The performance of the SPR-HAVP1-based assay was evaluated with serum samples. The antigen–antibody detection analysis by the variation of the initial and final dissociation values in the time (ΔRUd/t) was effective to show a significative difference between positive and negative serum. The possibility of determining a cutoff value for discrimination between positive and negative serum samples suggests the test’s feasibility for routine diagnosis, due to the ease of interpretation of the results generated. This reinforces the potential of the SPR-HAVP1 strategy as a new tool in serological monitoring for HA.

The relatively wide linearity range and high sensitivity observed from this study will be valuable for early diagnosis of acute hepatitis A, even during the period of infection, when the levels of anti-HAV IgM antibodies are low, thereby reducing the immunological window period of this infection.

Current gold standard immunoassays used for hepatitis A diagnosis employ a long incubation period for antibody–antigen association, indirect format [10,11], and can be relatively time-consuming when handling large sample sizes, as in an epidemic [15]. These shortcomings associated with conventional immunoassays can be overcome by the SPR-HAVP1-based assay, which allows for monitoring of antigen–antibody reactions in real time, without the use of conjugated developers, with high selectivity. SPR’s capacity to test large sample sizes, up to 768 samples per chip, together with the possibility of repeated use of the same chip, depending on the limit of regeneration cycles, demonstrates its special application for rapid and large-scale diagnosis of hepatitis A, thus contributing to fast actions to control and contain virus transmission during epidemic outbreaks and better knowledge of the epidemiological behavior of this etiologic agent. 

This study has limitations regarding the lack of information about the limit of detection and accuracy of the commercial ELISA used to validate the performance of the SPR-HAVP1 described here, which did not allow us to compare the efficacy between the assays. Another limitation was the small size of the serum samples tested. Thus, additional studies are needed to assess the serological status of persons living in hepatitis A-endemic areas with this simple test.

## 5. Conclusions

The richness of continuous and discontinuous B epitopes predicted for VP1 reinforces the ability of this protein as a serological marker in the diagnosis of HAV, motivating vanguard studies for new proposals for serological tests as proposed in this study. In this context, the SPR-HAVP1 platform presented here was a successful approach to detect human antibodies against HAV. This presents an open new field for SPR applications for coping with viral epidemics, such as hepatitis A.

## Data Availability

Not applicable.

## Supplementary Materials

The following are available online at https://www.mdpi.com/article/10.3390/s21093167/s1, Figure S1: Sonogram of analysis of the interaction between VP1 and bovine serum albumin (BSA). The ex-periments were conducted with 10 μg of BSA in a microflow of 10 μL/min. The interaction data is represented as a resonance response unit (RU) at time (seconds). These tests are representative of three repetitions; Figure S2: Serum concentration linearity in SPR assays. The tests were performed with positive and negative serum samples. Data show RU signals normalized (RU normalized) were propor-tional to the dilution (serum dilution), discriminated between positive and negative serum. These tests are representative of three repetitions.
