# Supplementary material for: Detection of Antibodies against Hepatitis A Virus (HAV) by a Surface Plasmon Resonance (SPR) Biosensor: A New Diagnosis Tool Based on the Major HAV Capsid Protein VP1 (SPR-HAVP1)
Source: Sensors (Basel). 2021 May 3;21(9):3167. doi: 10.3390/s21093167 (PMC8125114; doi:10.3390/s21093167)
Supplement: Supplementary file 1 [file sensors-21-03167-s001.zip › sensors-1056950-supplementary.pdf]

sensors

MDPI

Supplementary Materials

# Detection of Antibodies against Hepatitis A Virus (HAV) by a Surface Plasmon Resonance (SPR) Biosensor: A New Diagnosis Tool Based on the Major HAV Capsid Protein VP1 (SPR-HAVP1)

**Gabriel Menezes Costa dos Santos [1], Carlos Roberto Alves [2], Marcelo Alves Pinto [1], Luciane Almeida Amado Leon [1,*] and Franklin Souza-Silva [3]**

[1] Laboratório de Desenvolvimento Tecnológico em Virologia, Instituto Oswaldo Cruz, Fundação Oswaldo Cruz, Rio de Janeiro, RJ CEP 21040-900, Brazil; gabriel.santos@ioc.fiocruz.br (G.M.C.d.S.); marcelop@ioc.fiocruz.br (M.A.P.)

[2] Laboratório de Biologia Molecular e Doenças Endêmicas, Instituto Oswaldo Cruz, Fundação Oswaldo Cruz, Avenida Brasil, 4365, Manguinhos, Rio de Janeiro, RJ CEP 21040-900, Brazil; calves@ioc.fiocruz.br

[3] Centro de Desenvolvimento Tecnológico em Saúde, Fundação Oswaldo Cruz, Avenida, 4365 CEP 21040-900, Manguinhos, Rio de Janeiro, RJ, Brazil; Franklin.souza@cdts.fiocruz.br

* Correspondence: l_amado@ioc.fiocruz.br; Tel.:+55-21-2562-1876

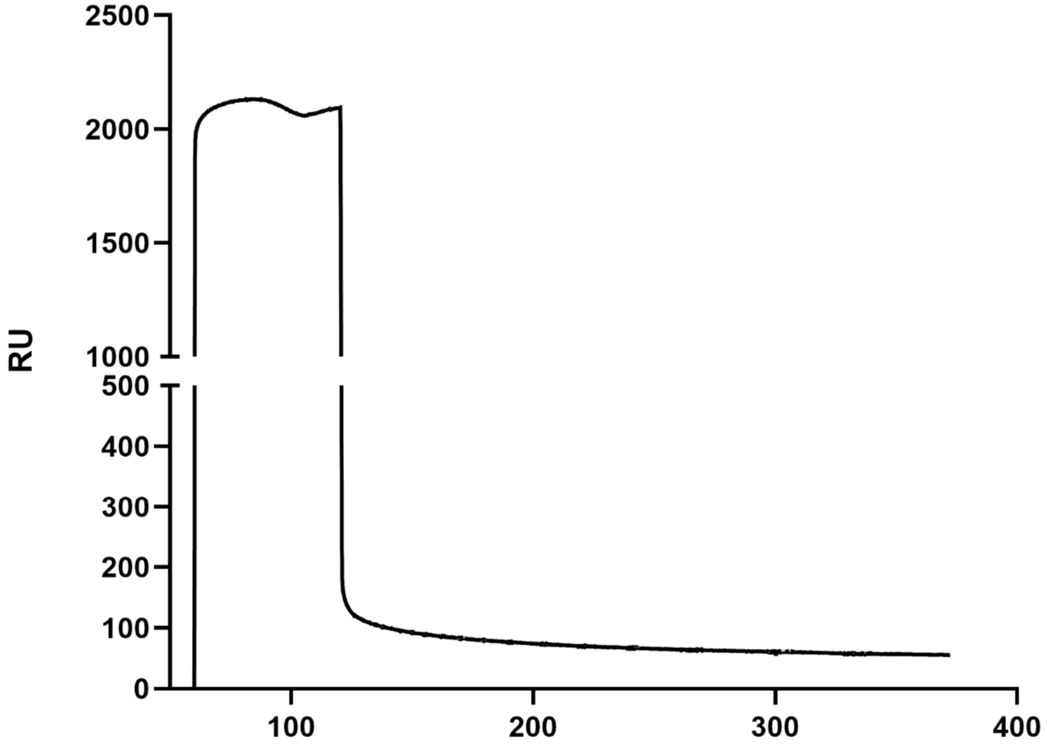


**Figure S1:** Sonogram of analysis of the interaction between VP1 and bovine serum albumin (BSA). The experiments were conducted with 10 µg of BSA in a microflow of 10 µL / min. The interaction data is represented as a resonance response unit (RU) at time (seconds). These tests are representative of three repetitions.

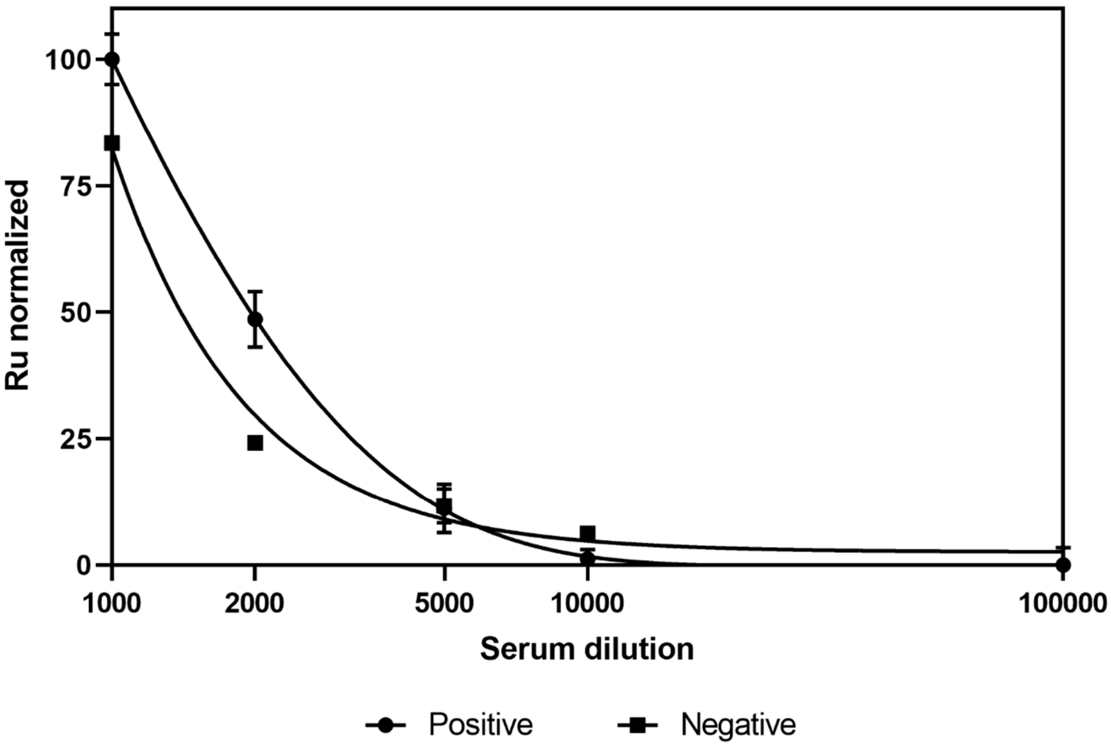


**Figure S2:** Serum concentration linearity in SPR assays. The tests were performed with positive and negative serum samples. Data show RU signals normalized (RU normalized) were proportional to the dilution (serum dilution), discriminated between positive and negative serum. These tests are representative of three repetitions.
